# The Utility of Circulating Tumor DNA (ctDNA) Monitoring in Cancer Patients Who Are Pregnant or Planning to Become Pregnant

**DOI:** 10.1155/2022/9412201

**Published:** 2022-03-15

**Authors:** Stacey A. Cohen, Anup Kasi, Nicole Hook, Michael Krainock, Griffin Budde, Allyson Koyen Malashevich, Jeffrey Meltzer, Russ Jelsema, Perry Olshan, Paul R. Billings, Alexey Aleshin, Andrew S. Poklepovic

**Affiliations:** ^1^University of Washington, Seattle, WA, USA; ^2^Fred Hutchinson Cancer Research Center, Seattle, WA, USA; ^3^University of Kansas Medical Center, Kansas City, KS, USA; ^4^Natera, Inc, Austin TX, USA; ^5^VCU Health System Massey Cancer Center, Richmond, VA, USA

## Abstract

The number of pregnant women with cancer is on the rise. These patients and their providers encounter complex medical management decisions. Standard-of-care systemic therapy and radiological imaging can impair fetal development and affect viability. Conversely, insufficient monitoring and treatment can lead to cancer progression, compromising the health of the patient. Personalized and tumor-informed circulating tumor DNA (ctDNA) testing (Signatera™, bespoke mPCR NGS assay) is a validated, noninvasive blood test that can accurately assess cancer progression and tumor response to treatment ahead of radiological imaging, across solid tumors. In this case series of four patients, we explore the clinical utility of longitudinal ctDNA testing in the medical management of pregnant patients with solid tumors, to aid in informed decision-making for patients and providers.

## 1. Introduction

Pregnant cancer patients face unique challenges, as standard-of-care treatments such as systemic therapy [1] and diagnostic imaging [2] can pose risks to the developing fetus. Although somewhat rare, affecting approximately 1 in 1000 pregnancies [3], the number of cases is increasing, partly due to trends in delayed childbearing [3] and diagnosis of cancer in younger individuals. These cases present complex medical management issues, including whether to continue the pregnancy, the modality, treatment frequency, the timing of diagnostic imaging, and the choice of antineoplastic therapy. The decision to initiate/continue treatment can have potentially severe impacts on fetal development. Improved predictive and prognostic biomarkers would allow for more informed decision-making. Advances in circulating tumor DNA (ctDNA) technology allow for the longitudinal monitoring of solid tumors without the need for invasive procedures and radiation [4]. ctDNA profiling can also aid in the decision to administer cancer treatments and to monitor the efficacy of these regimens, optimizing both cancer treatment and fetal safety [4]. Tumor-informed mPCR NGS-based ctDNA testing (Signatera™) has a high sensitivity and specificity for the postsurgical detection of molecular residual disease (MRD) that is predictive of relapse and clinical outcomes months before radiological detection, across cancers [5–7]. In this case series, we explore the ability of serial ctDNA testing to inform treatment decisions and pregnancy planning after a diagnosis of cancer, in four patients with solid tumors.

## 2. Personalized and Tumor-Informed ctDNA Testing

The personalized and tumor-informed ctDNA assay (bespoke mPCR NGS assay) is previously described [5]. Briefly, tumor and matched normal tissues were sequenced by whole-exome sequencing to identify up to 16 clonal, somatic, single-nucleotide variants (SNVs) for ctDNA monitoring in patient plasma samples. mPCR primers were designed and synthesized targeting the personalized SNVs. Plasma samples with at least two SNVs were considered ctDNA-positive. ctDNA concentration was reported in mean tumor molecules per milliliter of plasma (MTM/ml).

## 3. Case Presentation

### 3.1. Patient 1

**Figure 1:**
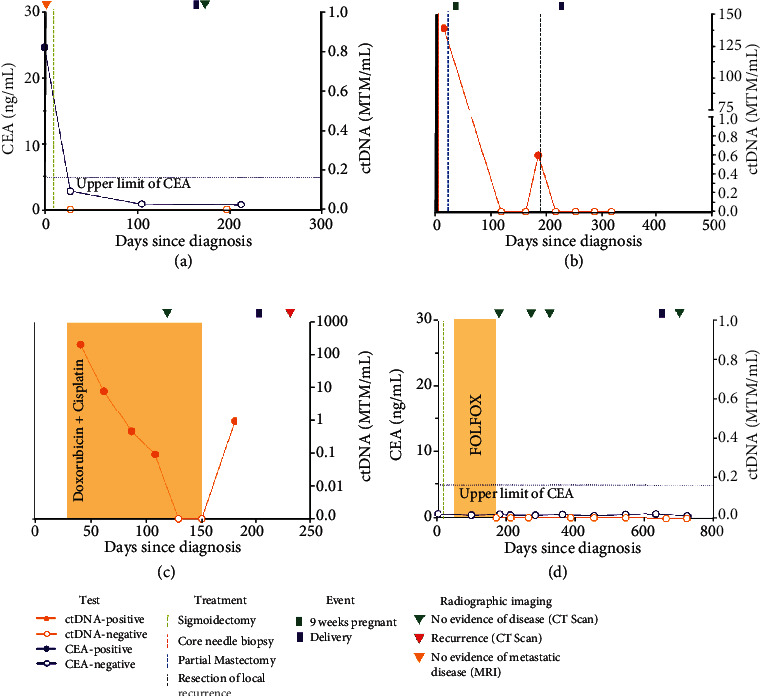
Use of tumor-informed mPCR NGS-based ctDNA testing in pregnant patients. (a–d) Individual patient clinical courses are represented. Circulating tumor DNA; CEA: carcinoembryonic antigen.

A 33-year-old pregnant woman at 17 weeks gestation presented with diarrhea and rectal bleeding. Flexible sigmoidoscopy revealed a mass in the sigmoid colon, and biopsy confirmed adenocarcinoma. CEA (carcinoembryonic antigen) was elevated at this time. CT of the chest and MRI of the abdomen and pelvis demonstrated no evidence of metastasis. The patient had proctosigmoidectomy with end colostomy. Pathology revealed stage IIA grade 2 moderately differentiated colorectal adenocarcinoma (pT3, pN0 0/34). Ancillary testing showed microsatellite stable disease. Adjuvant chemotherapy was not recommended, given the patient's pregnancy and low-risk, stage II status. The patient was started on surveillance with serial measurement of ctDNA and CEA. Surveillance CT scans were omitted due to the pregnancy. Five months after diagnosis, the patient delivered a healthy baby. A postpartum contrasted CT scan indicated no evidence of disease. Both ctDNA and CEA levels remained negative throughout the rest of the pregnancy (Figure 1(a)).

### 3.2. Patient 2

A 30-year-old pregnant woman presented at 5 weeks gestation with a left breast mass. Core needle biopsy revealed grade 3 T2N0 invasive ductal carcinoma. ER status was 15% positive; PR status and HER2 status were negative. Pre-surgical ctDNA was positive, at 139.22 MTM/mL. At 9 weeks gestation, the patient had partial mastectomy with negative margins. Despite standard-of-care recommendations, the patient chose not to receive further treatment during pregnancy and declined surveillance imaging. Postoperatively, ctDNA levels cleared and remained negative for two draws. Six-month post-surgery at 33 weeks gestation, ctDNA results returned positive, at 0.6 MTM/mL. At this time, the patient self-palpated a mass at the surgical site, subsequently confirmed as local recurrence. Following resection of the recurrent disease, ctDNA levels returned negative. Seven months after initial surgery and one month after ctDNA recurrence, the patient delivered a healthy baby. Post-delivery, serial ctDNA monitoring every 4-6 weeks demonstrated 4 negative tests (Figure 1(b)). Continued ctDNA testing and clinical breast exams are planned, and the patient has deferred all imaging.

### 3.3. Patient 3

**Figure 2:**
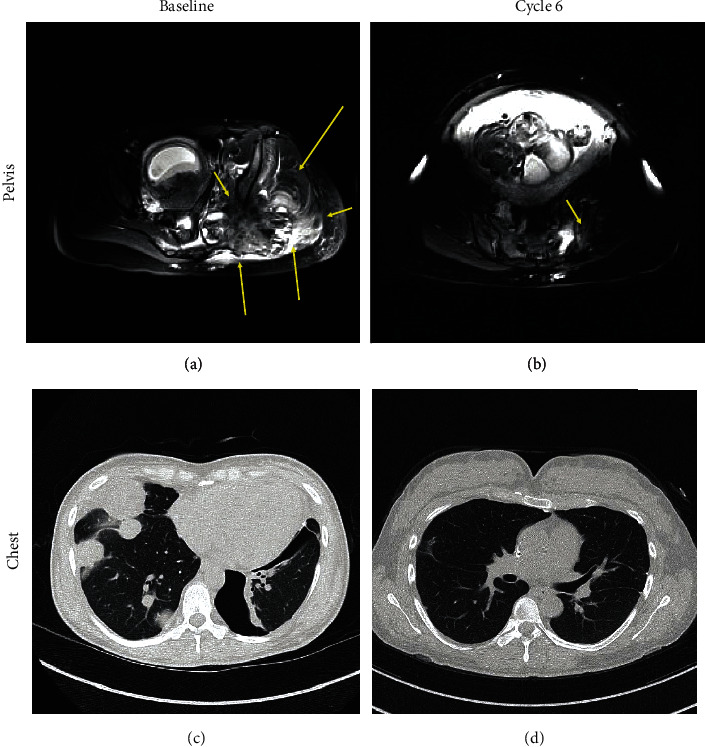
Computed tomography scans of (a, b) pelvis and (c, d) chest for case 3. CT scan was performed at baseline prior to starting treatment (a, c) and again after 6 cycles and 4.5 months of therapy (b, d). Resolution of lung metastases and pneumothorax is observed.

A 39-year-old pregnant woman at 9 weeks gestation presented with dyspnea on exertion. CT angiography demonstrated bilateral pneumothoraces and multiple pulmonary nodules. Concomitant hip pain prompted an MRI revealing a left gluteal mass. Pathology from the left upper lobe wedge was consistent with stage IVb (T3bN0M1b) osteosarcoma (OSA). OSA is typically treated with regimen of doxorubicin plus cisplatin, with or without methotrexate (MTX). The administration of MTX at a therapeutic dose could lead to fetal demise. The patient decided to limit frequency of imaging, have ctDNA levels monitored, and selected treatment of doxorubicin with cisplatin without MTX, starting at 11 weeks gestation. At this time, ctDNA was positive, at 213 MTM/mL. The patient decided that if early indicators showed a lack of response to doxorubicin and cisplatin, she would add MTX to her regimen and terminate the pregnancy. Following one cycle of systemic therapy, the patient exhibited a dramatic drop in ctDNA to 8.23 MTM/mL, and after 4 months of therapy, she cleared ctDNA. Concordantly, the patient achieved a dramatic radiographic response (Figure 2). The patient completed 6 full cycles of doxorubicin and cisplatin and delivered a healthy baby at term by cesarean section (Figure 1(c)). During the postpartum period, the patient relapsed, first testing ctDNA positive, which was later confirmed by imaging. The patient elected to start chemotherapy and is currently undergoing treatment for recurrent disease. The baby shows no signs of chemotherapy toxicity.

### 3.4. Patient 4

A 32-year-old woman presented with nausea, bloating, and hematochezia. She was found to have a circumferential, partially obstructing mass in the sigmoid colon. Biopsies revealed invasive moderately differentiated adenocarcinoma. Staging imaging revealed no evidence of distant metastasis, and the patient had sigmoidectomy. Pathology confirmed stage IIIb grade 2 moderately differentiated adenocarcinoma with invasion through the muscularis propria into subserosal adipose tissue (pT3) and involvement of lymph nodes (pN1b, 2/64). Ancillary testing was negative for *BRAF* mutations, microsatellite instability, with a CEA nonsecreting phenotype. Twelve cycles of adjuvant FOLFOX treatment were planned, but the patient developed neutropenia, fatigue, and neuropathy, refractory to dose reduction. Despite intolerance to the therapy, the patient was hesitant to end treatment early. In consideration of stopping adjuvant chemotherapy, CT imaging and ctDNA analysis were performed. Given no evidence of disease on imaging and a negative ctDNA result, the patient and provider elected to stop chemotherapy after the eighth cycle. CEA was normal at diagnosis and remained in the normal range throughout the patient's treatment. However, given the patient harbored a CEA nonsecreting tumor, this further justified the need for simultaneous ctDNA testing. Serial monitoring with ctDNA continued to demonstrate the absence of disease. Given her persistently negative results, the patient decided to become pregnant. To minimize impact to the fetus, CT scans were avoided during her pregnancy. ctDNA levels measured longitudinally remained negative throughout pregnancy, and the patient delivered a healthy baby. Two months following delivery, a contrasted CT scan indicated no evidence of disease and ctDNA/CEA remain negative (Figure 1(d)).

## 4. Discussion

Given the variation in effects of anticancer treatments across trimesters, pregnant cancer patients face time-critical decisions about their treatments. However, the aggressive therapies and standard-of-care imaging that lead to optimal oncologic outcomes pose serious risks to the developing fetus [1, 2]. For the first patient, who was diagnosed with colon cancer, postsurgical ctDNA-negative result supported her decision to avoid adjuvant chemotherapy and CT scans. In the second patient, who had declined radiation following partial mastectomy, ctDNA aided in the detection of a local, resectable recurrence of breast cancer. This case highlights the ability of ctDNA to indicate both local and distant recurrences in pregnant patients who choose to forego standard-of-care multimodal therapy. In the third patient, ctDNA was central to the selection of a safe and optimal chemotherapeutic regimen. Additionally, ctDNA served as a dynamic on-treatment response monitoring tool that allowed for the avoidance of imaging-related radiation during pregnancy. In the fourth case, ctDNA monitoring supported early cessation of adjuvant chemotherapy known to have significant side effects. Serial negative ctDNA results provided the patient with enough confidence to pursue pregnancy despite having not completed a full course of adjuvant chemotherapy. Furthermore, ctDNA testing allowed for the avoidance of CT scans during her subsequent pregnancy. As cancer continues to afflict patients of childbearing age [3], tools for noninvasive monitoring that can aid in the selection of patient-specific systemic therapeutic regimens are needed. Here, we demonstrate the ability of tumor-informed mPCR NGS-based ctDNA testing to serve as a noninvasive tool to monitor tumor progression, to aid in the decision to administer therapy, and to monitor response to therapy in pregnant patients with cancer. A major strength of the ctDNA methodology used in this study is that it is customized to measure 16 clonal single-nucleotide variants (SNVs) that are specific to the patient's tumor. At least two SNVs must be detected to trigger a positive call [5]. This approach leads to excellent sensitivity and specificity with no risk of false calls due to cell-free fetal DNA.

Pregnant patients with cancer are uncommon and often omitted from clinical trials. However, appropriate risk stratification is of the utmost concern for these patients. Overall, ctDNA has the ability to positively impact a pregnant patient's care in a noninvasive manner with minimal risk to the fetus and could be considered in cases where standard-of-care treatment may not best meet the needs of the patient.

## Data Availability

Data sharing requests should be submitted to the corresponding author (AP) for consideration. Access to deidentified data may be granted following submission of a written proposal and a signed data sharing agreement.
